# Evaluation and Management of Genitourinary Emergencies in Patients with Cancer

**DOI:** 10.1155/2021/4511968

**Published:** 2021-07-27

**Authors:** Demis N. Lipe, Phillip B. Mann, Rodrick Babakhanlou, Maria T. Cruz Carreras, A. Guido Hita, Monica K. Wattana

**Affiliations:** ^1^Department of Emergency Medicine, The University of Texas MD Anderson Cancer Center, Houston, TX, USA; ^2^Division of Urology, Department of Surgery, University of Texas at Houston, McGovern Medical School, Houston, TX, USA

## Abstract

**Background:**

Genitourinary emergencies in cancer patients are common. Most cancer treatments are administered in the outpatient setting, and patients with complications often visit the emergency department. However, there is no recent emergency medicine literature review focusing on genitourinary emergencies in the oncologic population. *Objective of the review*. To increase awareness of common genitourinary emergencies in patients with cancer and enable the prompt recognition and appropriate management of these conditions. *Discussion*. Genitourinary emergencies in patients with cancer require a multidisciplinary approach to treatment. The most common genitourinary emergencies in patients with cancer are related to infection, obstructive uropathy, hemorrhagic cystitis, and complications associated with urinary diversions. The treatment approach in patients with infections, including viral infections, is similar to those without cancer. Understanding the changes in the anatomy of patients with urinary diversions or fistulas can help with the management of genitourinary emergencies.

**Conclusions:**

Familiarization with the uniqueness of genitourinary emergencies in patients with cancer is important for emergency physicians.

## 1. Introduction

Genitourinary (GU) emergencies constitute a substantial proportion of the complications in patients with cancer, especially in those with urogenital malignancies [1], and they are often seen in the emergency department (ED) [2]. Some of these complications arise from direct tumor spread, while others are treatment-related complications resulting from surgery, radiotherapy, or chemotherapy [3–5]. Such emergencies, including obstructive uropathy, urinary tract infection (UTI), hemorrhagic cystitis (HC), postsurgical complications, rectovesical/vesicovaginal fistula, and complications of urinary diversion techniques [3], may increase patients' morbidity and mortality [6]. Therefore, it is important for emergency physicians to adequately recognize, evaluate, and manage these conditions, as the morbidity and mortality resulting from GU complications can be reduced by appropriate investigations and timely interventions [7, 8]. Accordingly, the purpose of this narrative, evidence-based review is to describe common urological emergencies in patients with cancer in order to increase the prompt recognition and appropriate management of these conditions.

## 2. Discussion

### 2.1. Methods

We performed a literature search of PubMed, EMBASE, and Google Scholar. The search included terms such as “urological emergencies,” “urological complications in cancer,” “prostate biopsy complications,” “nephrostomy complications,” “obstructive uropathy,” “ureteral obstruction,” “hemorrhagic cystitis,” “testicular cancer,” “penile-sparing surgery,” “penile cancer,” “scrotal cancer,” “radiation cystitis,” “bladder cancer,” “rectovesical fistula,” “vesicovaginal fistula,” and “recto-vaginal fistulas.” We also searched the references of the identified articles for relevant studies. We limited the search to full-text publications in English, Spanish, or German that dealt with disease in humans. The search resulted in over 500 articles, of which 65 were included after careful review and the removal of duplicates.

### 2.2. Obstructive Uropathy

GU, gynecologic, and retroperitoneal cancers can cause obstructive uropathy leading to acute kidney injury. Obstructive uropathy occurs when the normal flow of urine is blocked, resulting in hydroureter or hydronephrosis [9]. The presentation depends on several factors. Patients with acute obstructive uropathy may have flank pain, suprapubic pain, decreased urinary output, or elevated blood pressure [9]. Those with a partial instead of a complete obstruction of the bladder or ureter may have polyuria, nocturia, and urinary frequency. Those with a complete obstruction may present with anuria. A workup may show a normal creatinine level and glomerular filtration rate in patients with acute or partial obstructions or an abnormal creatinine level and glomerular filtration rate in patients with complete obstructions. A renal ultrasound may be helpful in evaluating for hydronephrosis; however, obstructions may be missed in patients with significant retroperitoneal disease or ureteral encasement [9, 10]. In such cases, a computed tomography (CT) scan can help further delineate the cause of the obstruction. Early detection and correction of any substantial underlying obstruction is essential for the preservation of renal function and minimization of cancer-therapy-related complications [11].

Patients with urinary obstruction often undergo placement of a percutaneous nephrostomy (PCN) tube or ureteral stent in the setting of ureteral obstruction or bladder outlet obstruction not amenable to direct bladder drainage. Postoperative complications associated with PCN placement include catheter displacement, bleeding, and urinary tract and skin infections [12–14]. Placement of a PCN tube has been reported to increase risk of mortality in patients with prostate cancer [14]. In the case of suspected catheter dislodgement, an abdominal x-ray, renal ultrasound, or CT scan can be performed to evaluate the catheter's positioning. Catheter patency can be assessed by simply flushing the catheter with sterile saline. Furthermore, if bleeding or infection is suspected, a CT scan of the abdomen (with contrast if the patient has adequate kidney function) can be done to evaluate for perinephric hematoma or abscess [10]. If a symptomatic (e.g., fever, severe pain, purulent drainage, malaise, altered mental status) UTI is suspected, a urine culture should be obtained, and antibiotics started. Patients with UTIs may need to have a new PCN tube placed by an interventional radiologist depending on the age and patency of the tube. In patients with stents, the stent failure rate can be as high as 58%, and those in whom stents fail will eventually need a PCN tube placement [15]. Stent-related complications include stent discomfort (e.g., bladder spasms), migration, and obstruction.

### 2.3. Bladder Complications

Emergencies involving the bladder are common in patients with cancer and can involve infection, bleeding, or complications associated with cancer treatment. The most common complications these patients face are UTIs, HC, complications related to surgical procedures (e.g., radical cystectomy), and bacillus Calmette–Guerin (BCG) therapy [3, 16–19].

#### 2.3.1. Urinary Tract Infections

Patients with cancer are more susceptible to UTIs and urosepsis than patients without cancer because of chronic immunosuppression from their disease or its treatment [16]. The most frequently isolated pathogens in this patient population are *Escherichia coli* (*E. coli*) and *Proteus*, *Enterobacter*, *Pseudomonas*, and *Candida* species. While fungal infections are rare, they can be frequently found in patients with urinary catheters. Similarly, tuberculosis infection of the urinary tract is not common but should be suspected in patients with sterile pyuria and persistent fever [3]. Clinically, patients with cancer present like patients without cancer and the treatment is similar. However, the presence of biofilm, which is often associated with urinary catheters, scar tissue, or an obstructive urinary tract, will generally require higher doses of antibiotics. This is a consideration to keep in mind when treating cancer patients who often have indwelling urinary catheters or history of urinary obstruction [16, 20].

#### 2.3.2. Hemorrhagic Cystitis

HC is a common bladder emergency in cancer patients, which can occur in patients who have undergone pelvic radiation therapy, in those who received specific types of chemotherapy, or due to infectious etiologies [16, 17, 21]. In the long term, radiation therapy causes endarteritis, chronic fibrosis, and scarring of the urothelium within the field of radiation. This in turn can cause sloughing of the urothelium and, subsequently, heavy bleeding. While HC can occur as early as 48–72 hours after conditioning regimen for stem cell transplant, the onset of chronic complications can occur up to 15 years after radiation therapy [16, 17, 21]. Furthermore, several cases have reported HC to occur with recreational drugs, environmental toxins, and even after ketamine use [21].

The drugs most associated with HC are systemic chemotherapeutic agents such as cyclophosphamide, ifosfamide, busulfan, idarubicin, and carboplatin; and intravesical chemotherapeutic agents such as doxorubicin, mitomycin, epirubicin, and BCG [16, 17, 21]. Patients with HC may present with typical UTI symptoms, such as frequency, urgency, and dysuria. In most patients, HC presents with recurrent mild bleeding with 7% to 53% having microscopic hematuria [22]. However, the frequency and quantity of hematuria are unpredictable, and in 20% of cases HC can be severe enough to warrant a blood transfusion [16]. Continuous bleeding from the bladder increases the risk of intravesical clot formation, which predisposes the patient to clot retention and bladder outlet obstruction. This is especially the case in older male patients with benign prostate hyperplasia [3].

About 5% to 25% of patients who have undergone hematopoietic SCT develop viral HC related to BK virus (BKV) [23, 24]. BKV infects more than 90% of the general population during childhood, and although it remains dormant in immunocompetent patients, it complicates the post-transplant courses of up to 25% of SCT patients [23]. Because it is associated with substantial morbidity, early recognition and treatment of viral HC is important. Patients who have undergone SCT and develop HC typically present with symptoms of HC approximately 2 months after SCT; macroscopic hematuria lasts approximately 2 to 4 weeks, during which up to 55% of patients experience clots [23]. Cytopenia and high-grade viremia are associated with worse outcomes and longer durations of illness [23].

Other viruses that have been implicated in HC are cytomegalovirus, JC polyomavirus, and the herpes simplex viruses [22]. Although cidofovir has been used for viral cystitis, its use remains controversial and the management remains supportive, rather than curative [22, 24]. Other infectious causes of HC include bacteria such as *E. coli, Staphylococcus saprophyticus, Proteus mirabilis,* and *Klebsiella* species. Fungal organisms include *Candida albicans, Cryptococcus neoformans, Aspergillus fumigatus*, and *Torulopsis glabrata* [22].

The initial approach to the treatment of a patient with HC is to assess the patient's hemodynamic status, as should be done for any bleeding patient. It is imperative to exclude other causes of hematuria, such as UTIs, urolithiasis, or medication-induced bleeding, such as that associated with the use of anticoagulants. A history should be taken and include questions about the patient's anticoagulant use, history of radiation therapy and chemotherapy, immunosuppression status, and recent instrumentation or procedures of the urological tract. The workup should include a complete blood count to evaluate platelet and hemoglobin counts, a coagulation panel, creatinine level, and a urinalysis with urine culture. Imaging modalities such as ultrasonography of the bladder, abdominal radiography, or CT imaging of the abdomen can clarify the source of the bleeding [3, 16]. Bedside ultrasound can be immediately useful in assessing the severity of clot and retention. Antibiotics to treat infections with the above-mentioned organisms should be initiated and reassessed once culture results are available [22].

The initial management in the ED should include the stabilization and resuscitation of hemodynamically unstable patients. Significant bleeding from the bladder, especially with clot retention, requires the insertion of a large bore (≥20 French), three-way bladder catheter with saline irrigation. The bladder should be hand irrigated with sterile fluid, removing all reducible clots prior to starting continuous bladder irrigation. If bleeding is intractable, the urology team may need to intervene with cystoscopy for clot evacuation and electrocoagulation [22]. Other secondary treatments may include intravesical installation of hemostatic agents, urinary diversion with nephrostomy tubes, selective embolization for refractory hemorrhage, and, as a last resort, surgical intervention to create a urinary diversion [22].

#### 2.3.3. Urinary Diversion

**Figure 1:**
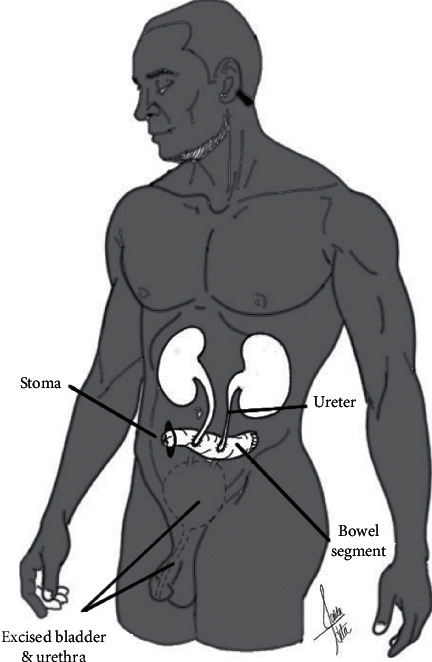
Continent cutaneous diversion. The ureters are attached to a pouch created by a bowel segment (ileum or colon). The pouch is then brought to the skin as a stoma. The urinary bladder and urethra are rendered nonfunctional by surgical removal or obliteration.

**Figure 2:**
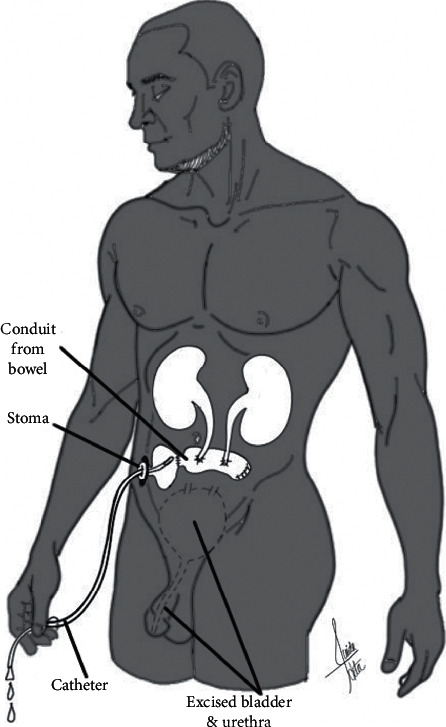
Noncontinent cutaneous diversion. Urine is drained from the ureters to a conduit constructed from the ileum or colon. The conduit is anastomosed to the abdominal skin surface, and urine is collected in an external appliance.

**Figure 3:**
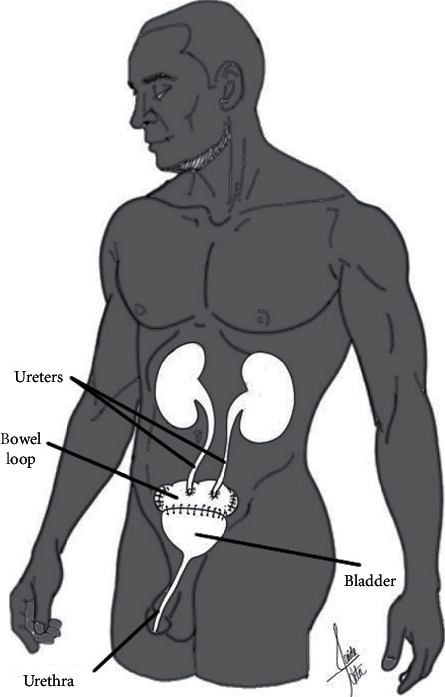
Orthotopic bladder substitution. A section of the bowel is used to reconstruct the bladder, allowing the use of the native urethral sphincter.

Besides being a last-resort treatment for HC, surgical intervention (e.g., radical cystectomy with urinary diversion) is the gold standard treatment for organ-confined, muscle-invasive bladder cancer. Various postcystectomy urinary diversion techniques are described in the literature and can be classified into three categories (Figures 1–3 ) [25, 26].

The literature describes complications occurring within the first 90 days after urinary diversion procedures as early complications, while those occurring after 90 days are considered late complications [26]. Early complications occur in between 20% and 57% of patients and are mainly attributable to the surgical procedure [26]. The most common early complications are gastrointestinal complications followed by infections and wound complications [26]. Because of the use of bowel for urinary diversions, patients may present to the ED with dehydration, paralytic ileus, obstructions, anastomotic leakages, or gastrointestinal bleeding. Infections commonly present as recurrent UTIs, urosepsis, intra-abdominal infections, or wound complications [25]. Late complications can be related either to the surgical procedure (e.g., stoma-associated complications, strictures of the ureteroenteric anastomosis, incisional hernias, urinary retention) or to long-term metabolic problems (e.g., electrolyte disturbances, vitamin deficiencies, bone disease, impaired hepatic metabolism, urolithiasis) [25, 27, 28]. The diagnostic workup in the ED requires a full laboratory investigation, urine testing, and imaging.

#### 2.3.4. Complications Associated with BCG Therapy

Intravesical BCG therapy has been used since the 1970s to treat bladder carcinoma in situ and bladder cancer that has not invaded the muscle. BCG is an attenuated, live mycobacterium, and its instillation into the bladder is believed to induce a local inflammatory reaction leading to the release of cytokines and induction of an antineoplastic immune response [18, 19].

Although BCG instillation is safe, both local and systemic side effects and complications can occur [19]. Mild symptoms usually arise within 48 hours after the second or third instillation and include cystitis, dysuria, low-grade fever, fatigue, malaise, or even hematuria [18]. Urine should be cultured to distinguish BCG cystitis from a bacterial UTI. Once a bacterial UTI has been excluded via a sterile culture, management of BCG cystitis is supportive, and patients can be treated with nonsteroidal anti-inflammatories, phenazopyridine, and/or antispasmodics. Should hematuria persist for more than 3 weeks, a cystoscopy is warranted to exclude a persistent cancer [18].

Severe symptoms are rare and usually occur within 48 hours of BCG instillation. These symptoms include high-grade fever, macroscopic hematuria, and irritation of the lower urinary tract and can involve solid organ systems and result in hemodynamic instability due to sepsis or allergic reactions, which requires immediate treatment [18]. Initial workup and empiric treatment should include urine and blood cultures for bacteria and acid-fast bacilli with administration of corticosteroids, broad spectrum antibiotics, and antituberculosis drugs [29–31].

### 2.4. Complications in Patients with Prostate Cancer

Diagnosis and treatment of prostate cancer involves various procedures that may lead to complications resulting in ED visits. Diagnostic workup of prostate cancer involves prostate biopsy—either transrectal or transperineal [32, 33], and treatment generally includes radiation, surgery, or systemic chemotherapy [34]. Transrectal prostate biopsy is most associated with early complications of urinary retention, lower urinary tract symptoms, hematuria, hematochezia, and hematospermia, although the feared complication is urosepsis. Other infections include asymptomatic bacteriuria, febrile UTI, acute bacterial prostatitis, orchitis, and epididymitis [33]. Fluoroquinolone resistance led to increasing rates of infectious complications, most commonly with uropathogenic *E. coli*; however, recent adoption of prophylaxis protocols has led to a decrease in urosepsis and prostatitis [32, 35–37]. In a recent comprehensive review, serious major complications after transrectal prostate biopsy requiring hospitalization range from 0.5% to 6.9% and have increased over time [32]. Although transrectal prostate biopsy remains the gold standard, transperineal prostate biopsy is increasing in popularity and has nearly eliminated infections and sepsis (0–0.2%); however, there is a slight increase risk of pain and urinary retention [32, 33, 37, 38].

Treatment for prostate cancer broadly includes radiation therapy and radical prostatectomy. Radiation therapy is either delivered externally or via implanted brachytherapy seed. External beam and hypofractionated radiotherapy are associated with a 0.8 to 9% rate of moderate to severe gastrointestinal (GI) and GU complications [39, 40]. GI toxicity is rare for brachytherapy, but GU toxicity ranges from 6 to 8%. With combined therapies, GI and GU toxicity can be as high as 18 and 31%, respectively [39]. ED presentations for such toxicities may include rectal bleeding, fistula, ulcer, gross hematuria from HC, or acute urinary retention due to voiding dysfunction or urethral stricture. Gross hematuria should be managed as previously discussed. Urethral stricture disease may be managed by inserting a smaller stiffer silicone (e.g., 12 French) catheter or may require urology consultation for urethral dilation—with or without cystoscopy—or suprapubic catheter placement.

Radical prostatectomy includes the surgical removal of the prostate, seminal vesicles, and proximal vas deferens—with or without pelvic lymph nodes—and vesicourethral anastomosis with catheter placement. The urinary catheter is placed to allow for bladder rest and urine drainage while the vesicourethral anastomosis heals. Typically, the catheter will stay in place for 3–7 days postsurgery [37]. Since 2012, 70% of radical prostatectomies have been performed with robotic assistance [41] and implementation of robotic technology has led to fewer ED visits than open surgery [42]. Short-term complications that may lead to an ED presentation include catheter malfunction, UTI, bladder neck contracture (i.e., urethral stenosis), and urinary retention, which combined have historically been reported around 7.5% [43]. Other complications include lymphocele, venous thromboembolism, infection, and urinary incontinence. Catheter-associated complications in the immediate postoperative period should be addressed with urology consultation.

### 2.5. Surgical Complications in Patients with Penile Cancer

Penile cancer is an uncommon malignancy in developed countries. Its incidence is 0.4% to 0.6% in North America, and it typically occurs during the sixth and seventh decades of life [44–46]. The incidence of penile cancer is higher in developing countries, where rates are as high as 10% to 20% [47]. Surgical intervention is the most common treatment modality for all stages of penile cancer, and the surgical method of choice depends on the lesion's stage, grade, and location. Most (80%) penile carcinomas involve the glans, coronal sulcus, or prepuce, and if cancer in these locations is identified early with no spread, superficial lesions can be treated with penile-sparing techniques such as local excision with or without circumcision, Mohs surgery, wide local excision, and glansectomy [44, 48, 49]. For more advanced stages of penile cancer, the standard surgical therapy involves wide excision with a partial or total penectomy. Depending on the extent of excision, skin grafts may be used to cover the defect. Reconstructive techniques and urethroplasties are used to restore function. Emasculation is the surgical procedure used for advanced tumors and involves the complete removal of the penis, scrotum, and testes. Cancers deep within the penis often require inguinal lymph node removal with sentinel lymph node biopsy, inguinal lymphadenectomy, or pelvic lymph node surgery, depending on the amount of lymph node involvement [45].

Penile cancer postsurgical complications range from easier-to-manage surgical site wound infections after penile-sparing procedures to more serious complications such as those from more invasive and extensive procedures. A study by Velazquez et al. [50] found the overall incidence rate of penectomy complications to be 19.7%; the most common complications were UTIs (3.0%), surgical site infections (3.0%), and bleeding requiring transfusion (3.9%). Other complications described in the literature include urethral stricture and Fournier gangrene [47]. If lymph node dissection within the groin is performed, the risk of surgical complications increases; infection is the most common complication followed by hematoma formation and thromboembolism [51].

Although patients presenting with surgical complications from penile cancer make up a small percentage of all patients seen in the ED, penile cancer surgery has a high rate of complications; thus, recognition and prompt management of those complications is imperative. Physicians should assess penile function by asking questions about urine output and pain with urination to determine if a urethral stricture or infection is present. A bedside bladder scan and postvoid residual urine test should be considered in patients having problems urinating. Clinicians should also assess for infection and the presence of venous thromboembolism and bleeding requiring transfusion. The physical examination requires an in-depth examination of the surgical site to look for possible infection and hematomas, and an examination of the lower extremities must be performed to determine if a workup for a potential deep vein thrombosis is also necessary. Emergency physicians should have a low threshold to use CT to image the surgical area to rule out infections and other complications. Management of surgical complications should involve consulting the urological service and treating the underlying conditions.

### 2.6. Surgical Complications in Patients with Testicular or Scrotal Cancer

Testicular cancer is a rare urological malignancy with a very favorable cure rate even in patients with advanced disease. The management of testicular tumors involves surgery regardless of the presence of metastasis; surgery typically involves a radical inguinal orchiectomy for removal of the tumor-containing testicle and the associated spermatic cord. Depending on the tumor stage and type, a retroperitoneal lymph node dissection to remove the lymph nodes around the aorta and inferior vena cava may also be performed [52, 53]. Like testicular cancers, primary scrotal cancers are rare, and surgical excision is recommended to excise the localized cancer. Closure of the wound is performed via primary or secondary intention or skin grafts [54].

Complications from radical inguinal orchiectomy include infection and scrotal or retroperitoneal hematoma secondary to postoperative wound hemorrhage. The most common complications from scrotal surgery include infection, hematoma, and persistent edema. Complications from retroperitoneal lymph node dissection can occur in up to 36% of patients, with major complications occurring in up to 19% of patients [55]. Short-term complications that emergency physicians should be aware of include wound infection, bowel ileus and obstruction, chylous ascites, renovascular injury, and neurologic injury. Complications of retroperitoneal lymph node dissection performed at the same time as orchiectomy affect less than 5% of patients; wound infection is the main one. Bowel obstruction is also possible and occurs in less than 2% of patients [56, 57]. Retroperitoneal lymph node dissection performed after chemotherapy has a higher complication rate [57, 58]. Given that patients undergoing this procedure may have comorbidities, larger tumors in difficult-to-access locations, and tumors that may adhere to vasculature and vital structures, the overall incidence rate is 20% to 30% and the mortality rate is 0.1% to 0.8% [57, 58].

The workup for postsurgical complications in patients with testicular or scrotal cancer is determined by the physician's degree of suspicion after a thorough history and physical examination. If infection is suspected, prompt administration of antibiotics is needed, and imaging of the affected area may be necessary. Signs and symptoms of ileus or obstruction will require abdominal imaging and surgical consultation. A hematoma diagnosis can be made via a scrotal ultrasound.

### 2.7. Fistulas

Rectovesical and vesicovaginal fistulas can complicate the course of illness in patients with cancer. They may result from chemotherapy, radiation damage, malignant tumor invasion, and pelvic surgery complications [59, 60]. Although these fistulas are infrequently life-threatening, they substantially reduce the patient's quality of life [61, 62]. Vesicovaginal fistulas, abnormal connections between the vagina and bladder, usually present with urinary leakage through the vagina, bleeding, and localized pain [59, 60]. Because most are caused by invasive tumors, surgical repair is the preferred treatment approach [61, 63]. However, the timing of vesicovaginal fistula treatment and the optimal surgical method are controversial [64]. Rectovesical fistula, an abnormal connection between the lumen of the colon and the bladder, is uncommon [62]. It remains a challenging condition owing to its complexity and rarity. Because it can lead to severe UTI, fecaluria, pneumaturia, and urine in the rectum, it seriously affects the patient's quality of life [65]. Surgical approaches for rectovesical fistula vary, and there is no consensus regarding the ideal approach for repair. Initial management in the emergency department for fistula involving the bladder should include insertion of an indwelling bladder catheter for urinary diversion. If the fistula defect is too large, percutaneous nephrostomy tubes may need to be placed.

## 3. Conclusion

GU emergencies in patients with cancer are common. The most common conditions are infection, obstructive uropathy, HC, and complications associated with the different types of urinary diversions. Being familiar with the characteristics of these GU emergencies can improve the quality of care provided by the emergency physician.
